# MtPT5 phosphate transporter is involved in leaf growth and phosphate accumulation of *Medicago truncatula*

**DOI:** 10.3389/fpls.2022.1005895

**Published:** 2022-09-06

**Authors:** Xue Wang, Chunxue Wei, Fei He, Qingchuan Yang

**Affiliations:** Institute of Animal Science, The Chinese Academy of Agricultural Sciences, Beijing, China

**Keywords:** phosphate, PHT1 transporter, MtPT5, *Medicago truncatula*, leaf growth, Pi accumulation

## Abstract

Phosphorus (P) is an indispensable mineral nutrient for plant growth and agricultural production. Plants acquire and redistribute inorganic phosphate (Pi) via Pi transporters (PHT1s/PTs). However, apart from MtPT4, functions of the *M. truncatula* (*Medicago truncatula*) PHT1s remain unclear. In this study, we evaluated the function of the PHT1 family transporter MtPT5 in *M. truncatula.* MtPT5 was closely related to AtPHT1; 1 in *Arabidopsis* (*Arabidopsis thaliana*) and GmPT7 in soybean (*Glycine max*). *MtPT*5 was highly expressed in leaves in addition to roots and nodules. Ectopic expression of *MtPT5* complemented the Pi-uptake deficiency of *Arabidopsis pht1;1Δ4Δ* double mutant, demonstrating the Pi-transport activity of MtPT5 in plants. When overexpressing *MtPT5* in *M. truncatula*, the transgenic plants showed larger leaves, accompanying with higher biomass and Pi enrichment compared with wild type. All these data demonstrate that MtPT5 is important for leaf growth and Pi accumulation of *M. truncatula* and provides a target for molecular breeding to improve forage productivity.

## Introduction

Phosphorus (P) is an essential mineral nutrient for plant growth. It plays various biological functions and is a major determinant of crop production (Raghothama, 1999). Inorganic phosphate (Pi) is the main form of P that can be absorbed by plant roots (Chiou and Lin, 2011; Vincent et al., 2012; López-Arredondo et al., 2014). The total P level in soil is high, but the soluble Pi is always limited due to its low mobility, as well as precipitation and fixation (Marschner and Rimmington, 1988; Yan et al., 2021). It has been reported that about 70% of cultivated land in the world is deficient in plant-available Pi. P is one of the limiting factors for cultivated plants (Smith and Schindler, 2009; Péret et al., 2011; López-Arredondo et al., 2013). To maintain crop yield, the usage of P fertilizer is increased annually (Dobre et al., 2014; Heuer et al., 2017). However, excessive fertilizer is not only a waste, but also leads to environmental issues (Zhang et al., 2013; Zak et al., 2018; Che et al., 2020).

Plants absorb and translocate Pi via Pi transporters (PHT1s/PTs; Versaw and Garcia, 2017; Dai et al., 2022). Hence, *PHT1* genes are potential targets for improving plants Pi efficiency and benefiting yields (Veneklaas et al., 2012). Most of the *PHT1* genes are root-specific, while some are highly expressed in the aerial part or nodules and involved in Pi redistribution (Chen et al., 2019; Wang et al., 2020). The firstly identified *PHT1* gene was *Pho84* cloned from *Saccharomyces cerevisiae* (Bun-ya et al., 1991). Then, numerous PHT1s have been identified in plants including *Arabidopsis* (*Arabidopsis thaliana*; Muchhal et al., 1996; Shin et al., 2004), *M. truncatula* (*Medicago truncatula*; Liu et al., 2008), rice (*Oryza sativa* L.; Liu et al., 2011), maize (*Zea mays* L.; Wang et al., 2020) and soybean (*Glycine max*; Chen et al., 2019). Nine PHT1 members were identified in *Arabidopsis* (Mudge et al., 2002). Among them, AtPHT1;1 and AtPHT1;4 play predominant roles in Pi uptake (Shin et al., 2004). GmPT7 was reported to be responsible for Pi uptake from soil into nodules and distribution to the fixation zones. Overexpression of *GmPT7* promotes plant growth and soybean yield (Chen et al., 2019). When overexpressing *OsPT1* in rice, transgenic plants accumulated more Pi in shoots and displayed increased tiller numbers compared with wild-type plants (Seo et al., 2008). Thus, investigation of the functions of PHT1s provides an efficient route for improving plants nutrient efficiency.

Currently, 11 PHT1s were identified in *M. truncatula* (Liu et al., 2008). Yeast kinetics assays showed that MtPT1, MtPT2, MtPT3, and MtPT4 are low-affinity Pi transporters. MtPT1, MtPT2, MtPT3, and MtPT5 share 84% sequence identity, but only MtPT5 displayed high affinity for Pi (Liu et al., 2008). MtPT4 is highly expressed in mycorrhizal roots, responsible for Pi acquisition from arbuscules (Harrison et al., 2002). It is also expressed in plant root tip in the absence of the arbuscular mycorrhizal (AM) fungus and modulates root branching, whereas it does not significantly affect Pi accumulation in plants without AM symbiosis (Cao et al., 2020). Recently, MtPT6 was reported to be involved in Pi uptake by heterologous expression of *MtPT6* in *Arabidopsis pht1;1* or *pht1;4* mutant. However, the role of MtPT6 in *M. truncatula* is unknown (Volpe et al., 2016). Information on the functions of PHT1s in *Medicago* is still limited.

In this study, we identified the role of MtPT5 in leaf growth and Pi accumulation of *M. truncatula. MtPT5* is highly expressed in roots, leaves, and nodules and is low-Pi inducible. MtPT5 can rescue the Pi-uptake deficiency of *Arabidopsis pht1;1Δ4Δ* double mutant, indicating the Pi transport activity of MtPT5 in plants. When overexpressing *MtPT5* in *M. truncatula*, the transgenic plants displayed larger leaf size and higher Pi content. These data demonstrate that MtPT5 plays important roles in *M. truncatula* vegetable growth and Pi nutrition.

## Materials and methods

### Plant materials and growth conditions

*Medicago truncatula* ecotype R108, *Arabidopsis thaliana* ecotype Wassilewskija ecotype (Ws) and *pht1;1Δ4Δ* mutant were used in this study. For germination of *M. truncatula,* seeds were placed on wet filter paper at 4°C for 2 days. Then, the imbibed seeds were transferred to chamber with illumination of 120 μmol m^−2^ s^−1^, temperature 24°C, and 16 h light/8 h dark photoperiod for 4 days. The seedlings were grown in 1/2 Hoagland or in soil for experiments. For nodulation, four-day-old seedlings were incubated with Sm1021 resuspended in 1/2 Hoagland, then transferred to soil and injected with Sm1021 every 2 days for 1 month. Different organs were harvested separately for RNA extraction. For *Arabidopsis* germination, the seeds were kept at 4°C for 2 days for imbibition, then transferred to medium with 200 μM arsenate or 1/2 MS under normal conditions (120 μmol m^−2^ s^−1^, 22°C, 16 h light/8 h dark).

### Measurement of Pi content

For *Arabidopsis*, the 20-day-old seedings grown on 1/2 MS medium were harvested for Pi content measurement. For *M. truncatula*, the top leaflets of 3-month-old plants grown in soil and 20-day-old seedlings grown in 1/2 Hoagland were collected. The measurement was assayed as described in the previous report (Ames, 1996). Briefly, different samples were collected and frozen in liquid nitrogen immediately. Pi was extracted in the buffer containing acetic acid at 42°C for 30 min. Pi concentration was measured at 820 nm wavelength using universal microplate spectrophotometer (BioTek Power Wave XS2). The Pi content was calculated based on the concentration and fresh weight of different samples.

### Plasmid construction and plant transformation

The full-length coding sequence (CDS) of *MtPT5* was cloned into *pTOPO-TA* Simple vector (Science Tool) for sequencing. Then the sequence-verified *MtPT5* CDS was constructed into *BamH* I-linearized *pCAMBIA1302* vector to generate *35S:MtPT5* plasmid via homologous recombination. The recombinant vector was used for plant transformation. For *Arabidopsis* (*pht1;1Δ4Δ* mutant), floral dip method was used as described (Clough and Bent, 1998) using *Agrobacterium tumefaciens* strain GV3101. The transformants were obtained on MS medium containing 50 mg/l hygromycin. For *M. truncatula*, the construct was introduced into R108 leaves via *Agrobacterium* EHA105-mediated transformation as described previously (Cosson et al., 2006). The transgenic *M. truncatula* plants were identified by PCR using vector-specific primers. T_2_ and T_3_ transgenic lines were used for *Arabidopsis* and *M. truncatula* separately in this study.

### qRT-PCR and RT-PCR analysis

For quantification of gene expression, total RNA was isolated using Eastep Super Total RNA Extraction KIT (Promega) and quantified by nanodrop. 1 μg RNA was used for reverse-transcription using the PrimeScript II 1st Strand cDNA Synthesis Kit (Takara). qRT-PCR was performed using 2 × EasyTaq® PCR SuperMix (TransGen Biotech) on CFX96 system (Bio-Rad). *MtActin11* was used to calculate the relative quantitative results for *M. truncatula*. The transcripts of *MtPT5* in R108, *pht1;1Δ4Δ* mutant and *pht1;1Δ4Δ/MtPT5* were tested by RT-PCR using cDNAs as templates. *EF1a* was amplified as a quantitative control.

### Sequence alignment and construction of phylogenetic tree

PHT1 amino acid sequences were obtained from NCBI^1^ and EnsemblPlants.^2^ Amino acid sequences were firstly aligned using CluxtalX. The neighbor-joining tree was conducted in MEGA5 using bootstrap method (900 replicates) on poisson model.

### Statistical analysis

Significant differences were determined by One-way ANOVA with Tukey test or Student’s *t*-test using SigmaPlot 12.5 software.

## Results

### Phylogenetic analysis of PHT1s from different species

**Figure 1:**
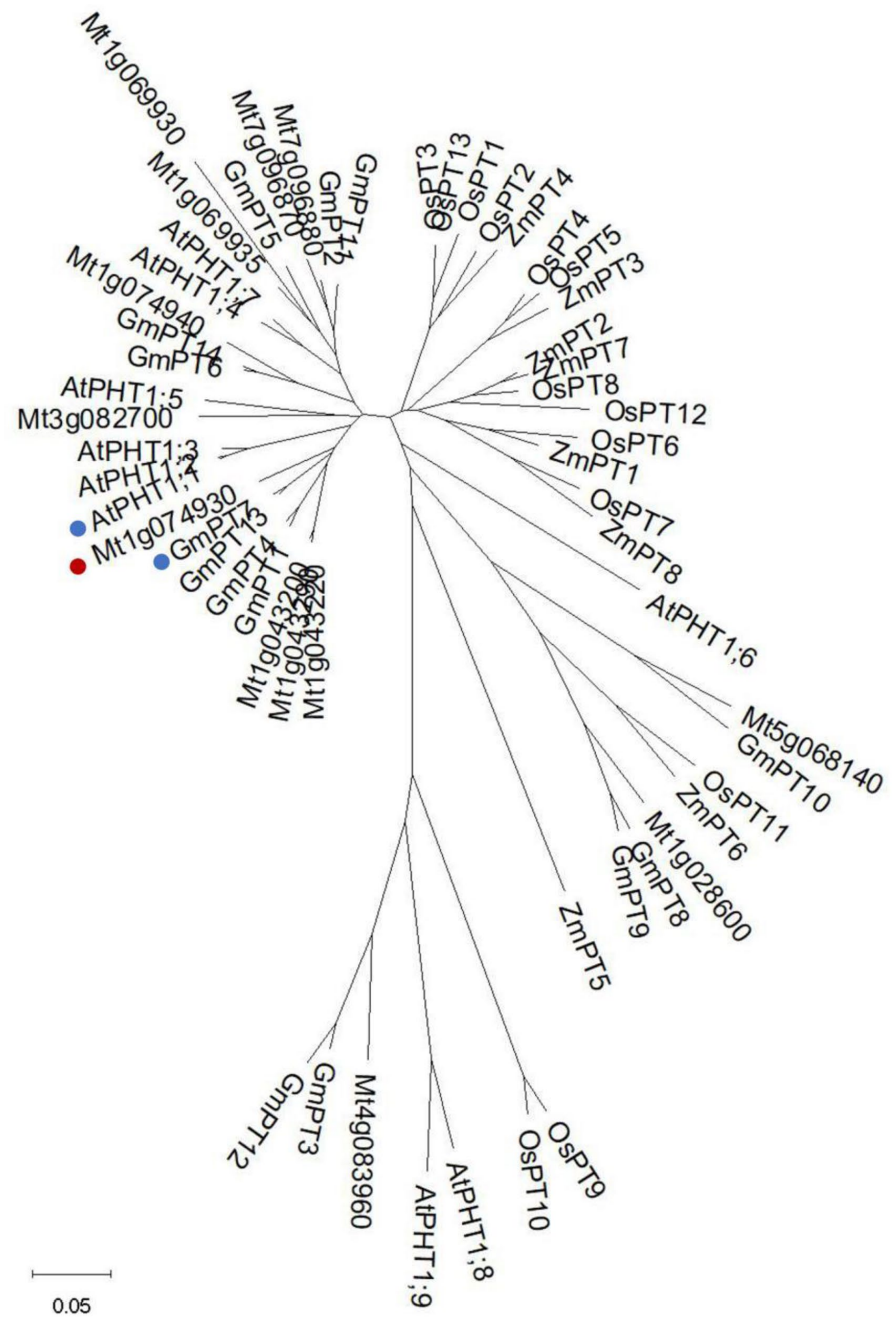
Phylogenetic analysis of PHT1s from different species. Phylogenetic tree of PHT1s from *Medicago truncatula*, *Arabidopsis*, soybean, maize, and rice. The tree was generated as described in materials and methods. Mt1g074930 (MtPT5) was labeled with a red spot. AtPHT1;1 and GmPT7 were labeled with blue spots. The bar shows 0.05 amino acid substitutions per site.

It has been reported that there are 11 PHT1 transporters in *M. truncatula* (Cao et al., 2020). We identified another two members (Mt4g083960 and Mt5g068140) by searching Ensemblplants.^3^ All members shared the common secondary structures with 11 predicted transmembrane domains (TM) separated by a large hydrophilic loop between TM6 and TM7 (Supplementary Figure S1). The signature GGDYPLSATIxSE (Karandashov and Bucher, 2005; Loth-Pereda et al., 2011) was identified and conserved among all MtPHT1s, except two of them. The signature of Mt1g069930 was modified with a Thr (T) replaced by a Val (V), and Mt1g074940 was modified with an Ala (A) replaced by a Ser (S; Supplementary Figure S1). The amino acid sequences of PHT1 proteins from *M. truncatula*, *Arabidopsis*, soybean maize and rice were used for constructing the neighbor-joining tree (Figure 1). The analysis showed that Mt1g074930 (MtPT5) was clustered phylogenetically to AtPHT1;1 and GmPT7, showing 80% and 86% amino acid sequence identities, respectively.

### Expression pattern of *MtPT5* in *Medicago truncatula*

**Figure 2:**
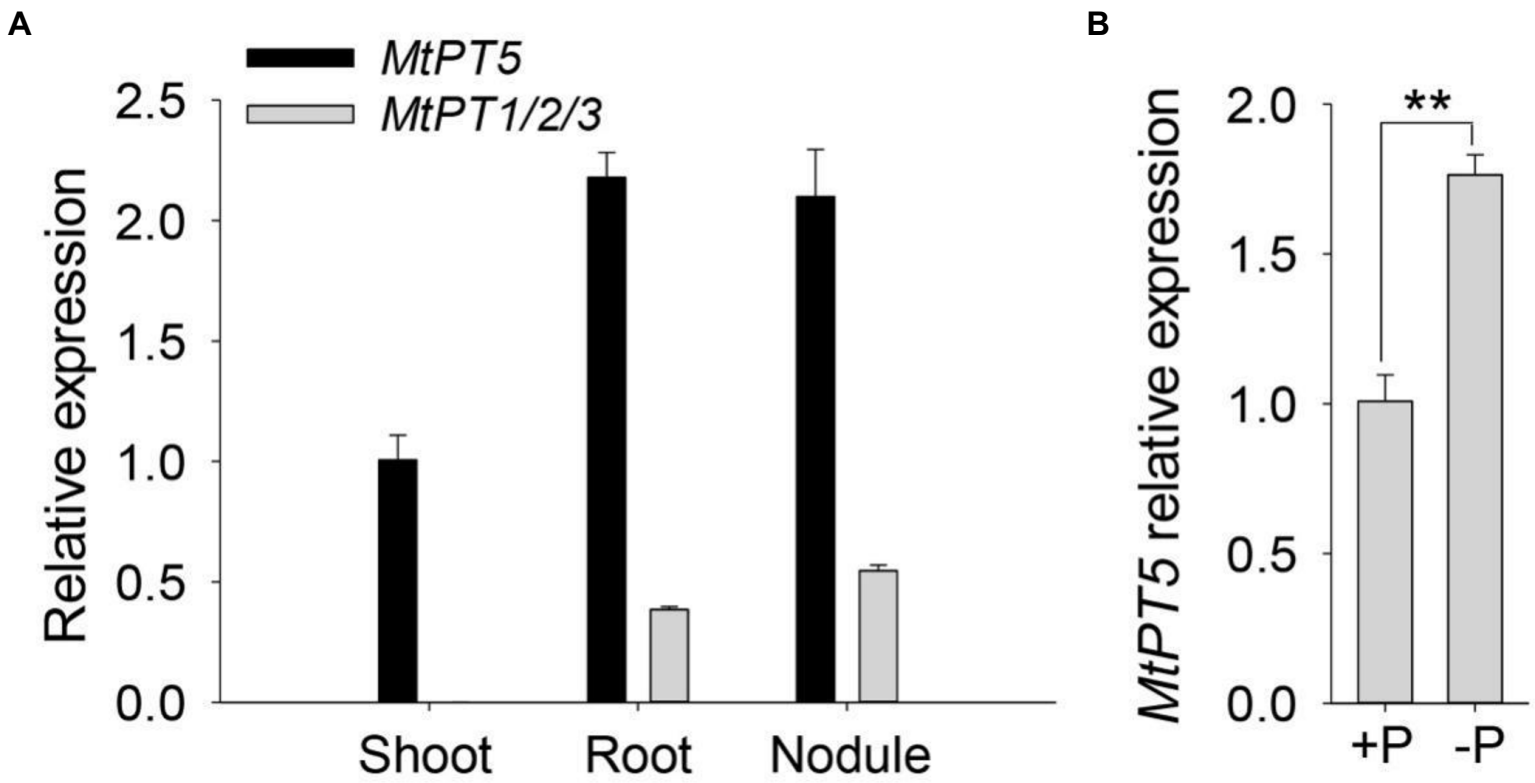
Expression profiles of *MtPTs* in *M. truncatula*. **(A)** qRT-PCR analysis of *MtPT1*/2/3 and *MtPT5* in different tissues of *M. truncatula.* Four-day-old wild-type seedlings (R108) were incubated with Sm1021 resuspended in 1/2 Hoagland and then transferred to soil and injected with Sm1021 every 2 days for 1 month. Shoots, roots, and nodules were harvested, respectively, for RNA extraction. Data represent mean ± SE (*n* = 3). **(B)** qRT-PCR analysis of *MtPT5* in wild-type seedlings (R108) during phosphate starvation. Four-day-old *M. truncatula* seedlings were transferred to hydroponic solution with Pi (+P) or solution without Pi (−P) for 5 days. The whole seedlings were used for RNA extraction. Data represent mean ± SE (*n* = 3). ** indicates significant difference at *p* < 0.01 (Student’s *t*-test).

MtPT1, MtPT2, and MtPT3 are paralogues of MtPT5 in *M. truncatula* (Liu et al., 2008). The coding sequences of *MtPT1*, *MtPT2*, and *MtPT3* share 97% identity. A single pair of primers were used to test the expression of these three genes. Quantitative RT-PCR (qRT-PCR) analysis showed that *MtPT1/2/3* was predominantly expressed in roots and nodules and nearly undetectable in shoots (Figure 2A). The transcription abundance of *MtPT5* was around four-to six-fold higher than that of *MtPT1/2/3* in the underground tissues. In addition, *MtPT5* was also highly expressed in shoots (Figure 2A). The expression pattern of *MtPT5* in the aerial part was further tested. qRT-PCR results showed that *MtPT5* was mainly expressed in leaves (Supplementary Figure S2). Pi starvation analysis showed that *MtPT5* was induced under Pi-deficient condition (Figure 2B), in accordance with the previous report (Liu et al., 2008). The expression profile suggests that MtPT5 probably have multiple functions in plants and crucial roles in leaves. Leaf is important for plant yield. Hence, MtPT5 was further analyzed in Pi nutrition and development of *M. truncatula*.

### MtPT5 rescued the Pi-uptake deficiency of *Arabidopsis pht1;1Δ4Δ* double mutant

**Figure 3:**
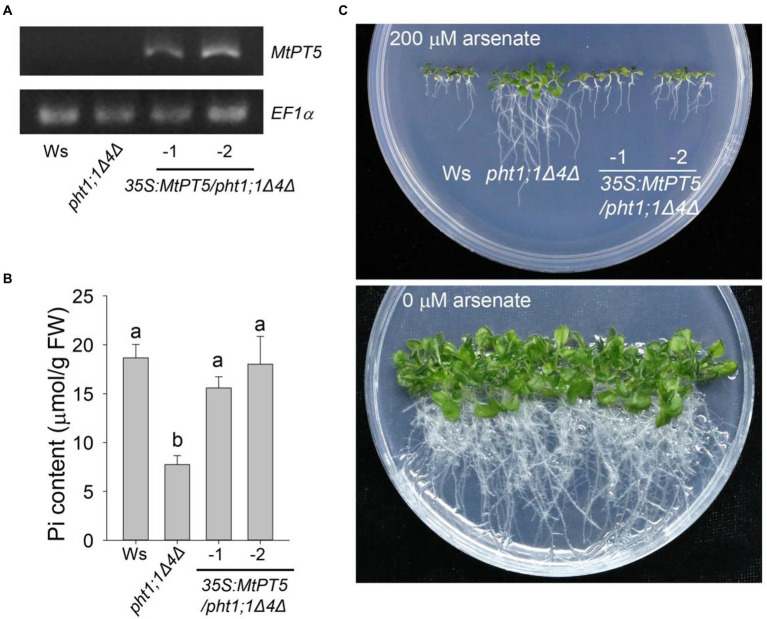
Ectopic expression of *MtPT5* enhanced Pi acquisition in *Arabidopsis*. **(A)** RT-PCR analysis of *MtPT5* in wild-type *Arabidopsis* (Ws), *pht1;1Δ4Δ* double mutant and *35S:MtPT5*/*pht1;1Δ4Δ* transgenic plants using *MtPT5*-specific primers. The housekeeping gene *EF1α* was used as an internal control. **(B)** Pi content measurement of wild-type *Arabidopsis* (Ws), *pht1;1Δ4Δ* double mutant and *35S:MtPT5*/*pht1;1Δ4Δ* transgenic plants. The whole seedlings grown on 1/2 MS for 20 days were collected for Pi extraction. Data represent mean ± SE (*n* = 3). Different letters indicate significant difference at *p* < 0.05 (One-way ANOVA, Tukey test). **(C)** Image of wild-type *Arabidopsis* (Ws), *pht1;1Δ4Δ* double mutant and *35S:MtPT5*/*pht1;1Δ4Δ* transgenic plants grown on 1/2 MS containing 200 μM arsenate or without arsenate for 20 days.

AtPHT1;1 and AtPHT1;4 are two major Pi transporters in *Arabidopsis*. The double mutant *pht1;1Δ4Δ* displayed dramatic reduction in Pi uptake capacity and Pi content compared with wild type (Shin et al., 2004). To examine the Pi uptake activity of MtPT5 in plants, the coding sequence of *MtPT5* driven by 35S promoter (*35S:MtPT5*) was introduced into *pht1;1Δ4Δ*. Two independent transgenic lines, *35S:MtPT5/pht1;1Δ4Δ-1* and *35S:MtPT5/pht1;1Δ4Δ-2*, were used in this study. RT-PCR analysis showed that the *MtPT5* transcripts were present in the two transgenic lines and not detectable in wild type (Ws) and *pht1;1Δ4Δ* mutant (Figure 3A). The fresh weight (FW) measurement showed that loss of *PHT1;1* and *PHT1;4* led to about 27% reduction in *pht1;1Δ4Δ* mutant biomass compared with wild type, similar to the previous report (Shin et al., 2004). Meanwhile, the biomasses of *35S:MtPT5/pht1;1Δ4Δ* transgenic lines could be rescued to the level of wild type (Supplementary Figure S3). This indicates that MtPT5 can rescue the morphological defects of *pht1;1Δ4Δ* mutant. Next, we tested the Pi contents in different genotypic *Arabidopsis* seedlings grown under Pi-sufficient condition (1/2 MS). Pi content in *pht1;1Δ4Δ* mutant was significantly reduced compared with wild type, while the two overexpression lines exhibited similar Pi contents with wild type (Figure 3B). These data suggest that MtPT5 can complement the Pi-uptake deficiency of *pht1;1Δ4Δ* mutant.

Arsenate is a toxic metalloid structurally analogous of Pi and is transported into root cells mainly via PHT1 transporters (Catarecha et al., 2007; Castrillo et al., 2013; Wang et al., 2014). Phenotypes of wild type, *pht1;1Δ4Δ* mutant and *35S:MtPT5*/*pht1;1Δ4Δ* transgenic plants were compared on the medium with or without arsenate. When grown on the medium with 200 μM arsenate, the *pht1;1Δ4Δ* mutant showed an arsenate-tolerant phenotype as previously reported (Shin et al., 2004), while the wild type and *35S:MtPT5*/*pht1;1Δ4Δ* seedlings were hypersensitive to arsenate with dramatically shorter roots and smaller shoots (Figure 3C). Taken together, these data indicate that MtPT5 has Pi transport capacity and positively modulates Pi uptake in plants.

### MtPT5 promotes leaves growth of *Medicago truncatula*

**Figure 4:**
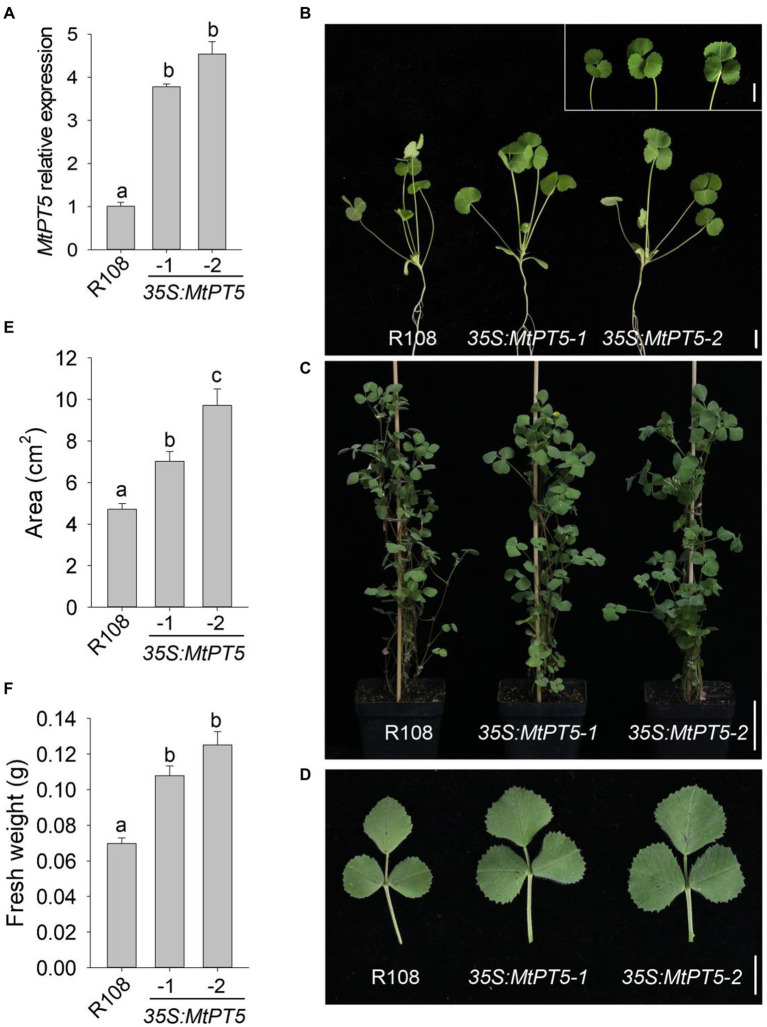
Overexpression of *MtPT5* promotes leaves growth of *M. truncatula.* **(A)** qRT-PCR analysis of *MtPT5* in wild-type *M. truncatula* (R108) and *MtPT5*-overexpressing plants (*35S:MtPT5-1* and *35S:MtPT5-2*). Data represent mean ± SE (*n* = 3). Different letters indicate significant difference at *p* < 0.05 (One-way ANOVA, Tukey test). **(B–D)** Phenotypic comparation of wild type (R108) and *MtPT5-*overexpressing plants. **(B)** Four-day-old seedlings were transferred to 1/2 Hoagland and grown for 20 days. Inset is leaves detached from the indicated plants. Bars = 1 cm. **(C)** Plants grown in soil for 3 months. Bar = 5 cm. **(D)** Top leaflets detached from **(C)**. Bar = 1 cm. **(E)** Quantification of leaf areas. The fourth leaf of wild type (R108) and *MtPT5-*overexpressing plants shown in **(B)** were taken for quantification. Data represent mean ± SE (*n* = 8). Different letters indicate significant difference at *p* < 0.05 (One-way ANOVA, Tukey test). **(F)** Fresh weight of four expanded leaves detached from (B). Data represent mean ± SE (*n* = 8). Different letters indicate significant difference at *p* < 0.05 (One-way ANOVA, Tukey test).

Given that *MtPT5* was induced by low-Pi stress, two independent *MtPT5*-overexpressing lines, *35S:MtPT5-1* and *35S:MtPT5-2*, were generated to examine the physiological role of *MtPT5* in *M. truncatula*. qRT-PCR analysis showed that both *MtPT5-*overexpressing lines had significantly increased *MtPT5* transcripts compared with wild-type *M. truncatula* (Figure 4A). We performed phenotypic tests on wild type and *MtPT5-*overexpressing plants. In both hydroponic culture and soil pots, the *MtPT5*-overexpressing lines displayed larger leaves compared with wild type (Figures 4B–D). Quantifications of leaf area confirmed this phenotype (Figure 4E). Meanwhile, leaf biomasses of the *MtPT5*-overexpressing plants were significantly higher than that of wild type (Figure 4F). These morphological traits indicate that overexpression of *MtPT5* promotes leaves growth in *M. truncatula*.

### Overexpression of *MtPT5* enhances Pi accumulation of *Medicago truncatula*

**Figure 5:**
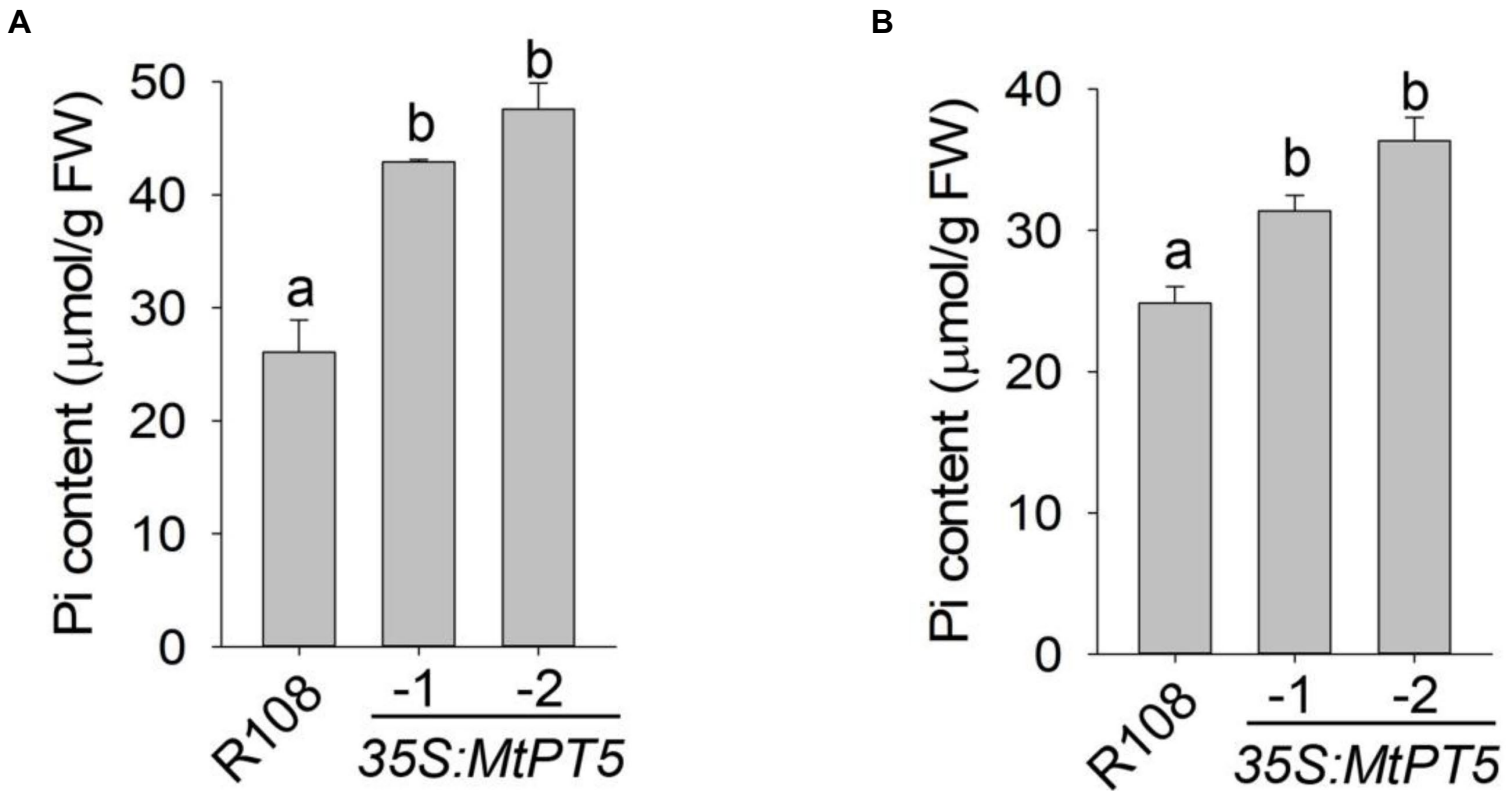
Overexpression of *MtPT5* enhances Pi accumulation of *M. truncatula.* **(A)** Pi content in top leaflets of wild type (R108) and *MtPT5-*overexpressing plants grown in soil for 3 months. Data represent mean ± SE (*n* = 3). Different letters indicate significant difference at *p* < 0.05 (One-way ANOVA, Tukey test). **(B)** Pi content of wild type (R108) and *35S:MtPT5*-overexpressing plants. Four-day-old seedlings were transferred to 1/2 Hoagland and grown for 20 days, then the whole plants were taken for Pi extraction. Data represent mean ± SE (*n* = 5). Different letters indicate significant difference at *p* < 0.05 (One-way ANOVA, Tukey test).

To explore the function of MtPT5 in *M. truncatula* Pi nutrition, we measured the Pi content in leaves of wild type and *MtPT5-*overexpressing lines. The top leaflets of plants grown in soil for 3 months were collected for Pi extraction. The measurement showed that relative to wild type, the Pi content in *MtPT5*-overexpressing plants increased dramatically, especially in *35S:MtPT5-2* line (Figure 5A). The Pi contents of whole plants were also measured. Various plants grown in hydroponic culture for 20 days were harvested. The *MtPT5*-overexpressing plants showed significantly higher Pi contents than wild type (Figure 5B). Taken together, these data indicate that overexpression of *MtPT5* enhances Pi accumulation in *M. truancatula.*

## Discussion

Phosphorus (P) is a major determinant of agriculture production. Plants absorb Pi via PHT1 transporters (Harrison et al., 2002), while some of them participate in Pi translocation and remobilization among different organs and tissues (Chang et al., 2019; Wang et al., 2020). It provides opportunities for improving crop performance by studying the functions of PHT1s (Chen and Liao, 2017; Han et al., 2022). Currently, 11 PHT1s have been found in *M. truncatula.* MtPT4 is responsible for Pi acquisition from mycorrhiza and plant root branching (Harrison et al., 2002; Volpe et al., 2016). MtPT6 was reported to promote Pi acquisition in *Arabidopsis* (Cao et al., 2020). Except for MtPT4, the functions of other PHT1s in *M. truncatula* are still unclear. In this study, we uncovered that Pi transporter MtPT5 plays an important role in leaf growth and Pi accumulation in *M. truncatula*.

### Analysis of different PHT1s

We found two more PHT1s (Mt4g083960 and Mt5g068140) in *M. truncatula* by searching Ensemblplants.^4^ Alignment analysis showed that the 13 PHT1s all contained 12 predicted transmembrane domains, in accordance with the previous report (Pedersen et al., 2013). To choose one member of PHT1 family for further study in *M. truncatula*, phylogenetic tree was firstly constructed using PHT1s from *M. truncatula*, *Arabidopsis*, soybean, maize, and rice. The analysis showed that MtPT5 was closely related to AtPHT1;1 and GmPT7. AtPHT1;1 is an essential Pi transporter in *Arabidopsis*. Under Pi sufficient condition, the mutation of *PHT1;1* leads to about 50% reduction of Pi uptake compared with wild-type plants. The Pi uptake of *pht1;1Δ4Δ* mutant reduces about 75% compared with wild type (Shin et al., 2004). GmPT7 is a nodule-located Pi transporter and responsible for the direct Pi acquisition from soil and Pi translocation from nodules to plant. Overexpression of *GmPT7* improves shoot P content, nitrogen (N) content and soybean yield (Chen et al., 2019). The phylogenetic analysis indicates that MtPT5 probably have essential roles in *M. truncatula* Pi nutrition. The amino acid sequence of MtPT5 shared 84% identity with MtPT1, MtPT2 and MtPT3, whereas MtPT5 displayed an opposite affinity for Pi (Liu et al., 2008). This indicates the multiple functions of different PHT1s in Pi utilization even though PHT1s share high amino acid identities.

### Function of MtPT5

MtPT5 was reported to be a membrane-located high-affinity Pi transporter (Liu et al., 2008). To examine the Pi uptake activity of MtPT5 in plants, the coding sequence of *MtPT5* driven by 35S promoter (*35S:MtPT5*) was constructed and introduced into *Arabidopsis* double mutant *pht1;1Δ4Δ.* Phenotypic analysis showed that *pht1;1Δ4Δ* mutant displayed an arsenate-tolerant phenotype, while the wild type and *35S:MtPT5/pht1;1Δ4Δ* plants displayed arsenate-toxic phenotype. Pi content measurement showed that the Pi content in *pht1;1Δ4Δ* was significantly reduced compared with wild type, in accordance with previously reported (Shin et al., 2004). The Pi contents in *35S:MtPT5/pht1;1Δ4Δ* transgenic lines were rescued to the level of wild type. Taken together, these data demonstrate that MtPT5 has the Pi-transporter activity in plants.

To identify the function of *MtPT5* in *M. truncatula*, two independent *MtPT5*-overexpressing lines (*35S:MtPT5-1* and *35S:MtPT5-2*) were generated with significantly higher *MtPT5* transcript levels. The *MtPT5*-overexpressing lines displayed larger leaves compared with wild type, and the leaf biomasses of the transgenic plants were increased dramatically. The Pi contents of top leaflets and whole plant in *MtPT5*-overexpressing lines were much higher than that in wild-type plants. These data demonstrate that overexpression of *MtPT5* enhances *M. truncatula* leaf growth and Pi accumulation.

## Conclusion

Expression analysis showed that *MtPT5* was highly accumulated in shoots, roots and nodules. Previous reports demonstrated that *ZmPT7*, which is expressed in both roots and leaves, participates in Pi acquisition and redistribution in maize (Wang et al., 2020). GmPT7, located to nodules, is responsible for the direct Pi uptake from soil and translocation to fixation zones (Chen et al., 2019). The expression profile of *MtPT5* suggests that it probably have multiple functions in Pi nutrition. In this study, we demonstrate that MtPT5 plays a vital role in Pi accumulation, and overexpression of *MtPT5* promotes the leaf growth of *M. truncatula* dramatically. Leaf size is a vital trait to improve the yield and quality of forage, such as legume alfalfa (*Medicago sativa* L.) (Warman et al., 2011; Zhang et al., 2019). It was reported that about 70% protein of alfalfa is stored in leaves, while the cellulose content in leaves is only 1/3 of that in stems (Yang et al., 2016). Hence, our study provides a clue for elevating alfalfa Pi efficiency and genetic breeding.

## Data availability statement

The original contributions presented in the study are included in the article/Supplementary material, further inquiries can be directed to the corresponding author.

## Author contributions

XW and QY planned and designed the research. XW and CW performed the experiments. XW wrote the manuscript. QY supervised this work and reviewed the manuscript. All authors contributed to the article and approved the submitted version.

## Funding

This work was supported by China Postdoctoral Science Foundation (2021M693441).

## Conflict of interest

The authors declare that the research was conducted in the absence of any commercial or financial relationships that could be construed as a potential conflict of interest.

## Publisher’s note

All claims expressed in this article are solely those of the authors and do not necessarily represent those of their affiliated organizations, or those of the publisher, the editors and the reviewers. Any product that may be evaluated in this article, or claim that may be made by its manufacturer, is not guaranteed or endorsed by the publisher.

## Footnotes

^1^ https://www.ncbi.nlm.nih.gov/

^2^ http://plants.ensembl.org/Medicago_truncatula/Info/Index

^3^ http://plants.ensembl.org/Medicago_truncatula/Info/Index

^4^ http://plants.ensembl.org/Medicago_truncatula/Info/Index
