# Supplementary material for: MtPT5 phosphate transporter is involved in leaf growth and phosphate accumulation of Medicago truncatula
Source: Front Plant Sci. 2022 Sep 6;13:1005895. doi: 10.3389/fpls.2022.1005895 (PMC9485599; doi:10.3389/fpls.2022.1005895)

Supplementary Material

**MtPT5 phosphate transporter is involved in leaf growth and phosphate accumulation of *Medicago truncatula***

**Xue Wang, Chunxue Wei, Fei He, Qingchuan Yang***

Institute of Animal Science, the Chinese Academy of Agricultural Sciences, Beijing 10019, China.

***Correspondence:**

Qingchuan Yang

qchyang66@163.com


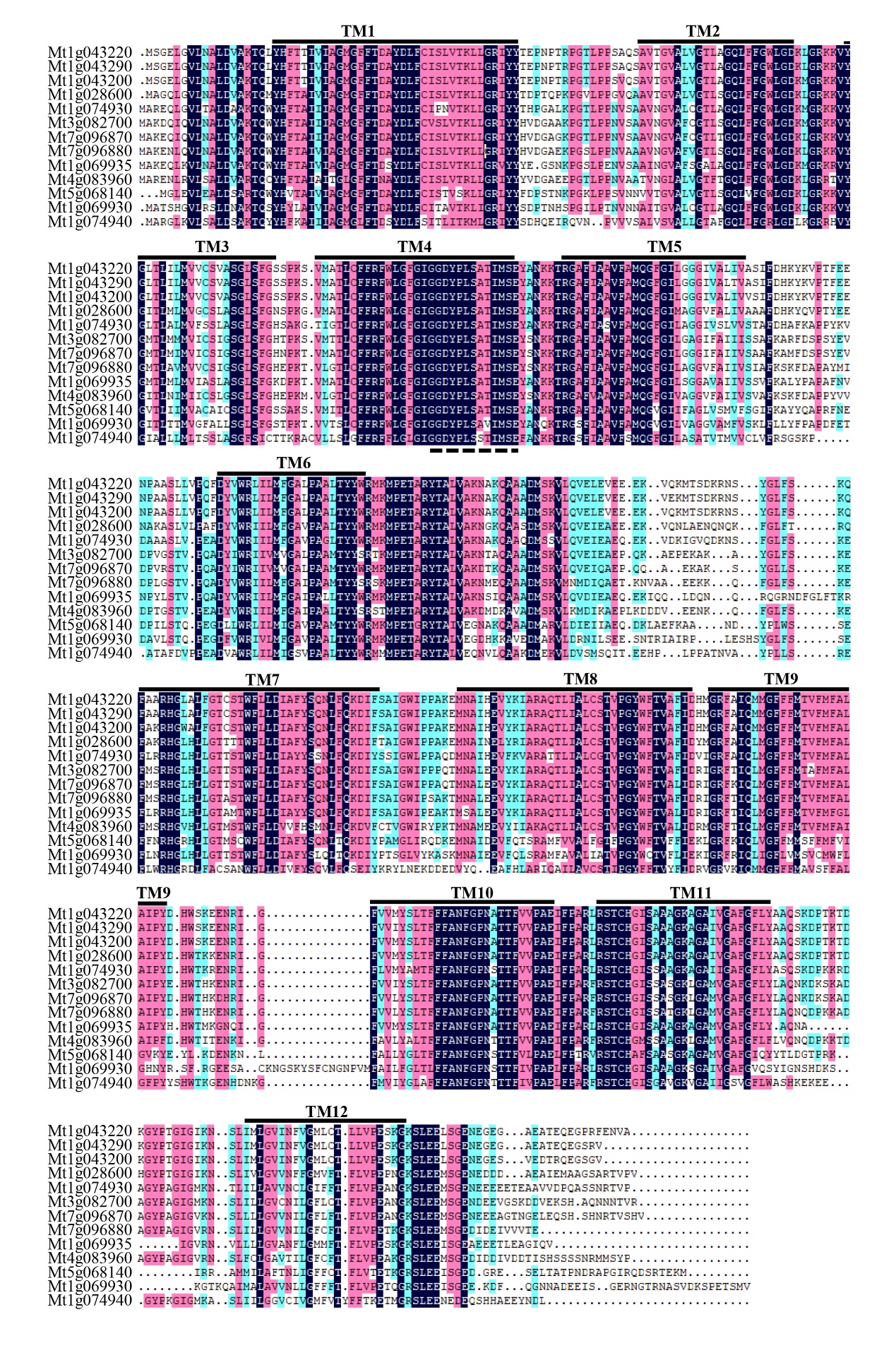


**Fig. S1 Alignment of amino acid sequences of PHT1s in *M. truncatula.***

Thirteen PHT1 family members from *M. truncatula* including MtPT1 (Mt1g043220), MtPT2 (Mt1g043290), MtPT3 (Mt1g043200), MtPT4 (Mt1g028600), MtPT5 (Mt1g074930), MtPT6 (Mt3g082700), MtPT7 (Mt7g096870), MtPT8 (Mt7g096880), MtPT9 (Mt1g069935), Mt5g068140, Mt4g083960, Mt1g069930 and Mt1g074940. Amino acids were aligned using DNAMAN program. The transmembrane domains (TM) are predicted using TMHMM and marked with lines. The conserved amino acids were shaded with different colors.


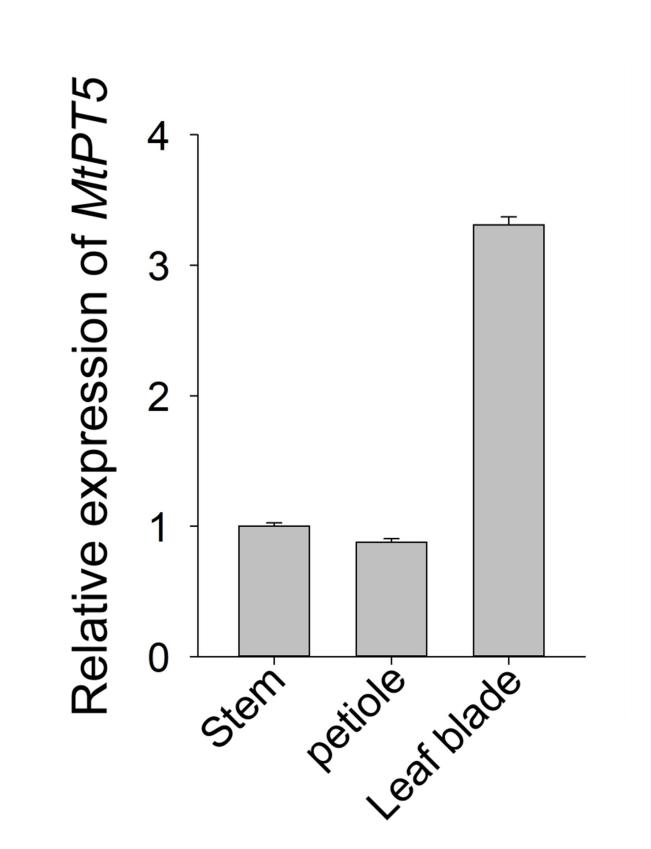


**Fig. S2 Expression pattern of *MtPT5* in selected tissues of *M. truncatula***

R108 plants grown in 1/2 Hoagland for one month were used for expression analysis.

The three indicated tissues were collected for RNA extraction. Data represent mean ± SE (n = 3).

**
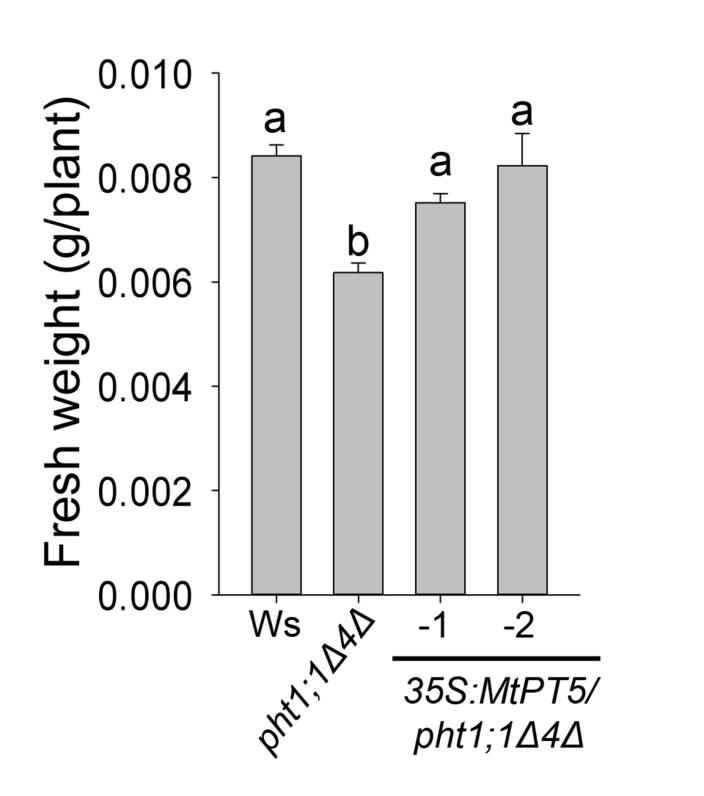
Fig. S3 Fresh weight of wild-type *Arabidopsis* (Ws), *pht1;1Δ4Δ* mutant and *35S:MtPT5/pht1;1Δ4Δ* transgenic plants.**

The seedlings grown on 1/2 MS for twenty days were used for shoot biomass statistics.

Data represent mean ± SE (n = 3). Different letters indicate significant difference at *P* < 0.05 (One-way ANOVA, Tukey test).

**Table S1 Primers used in this study**


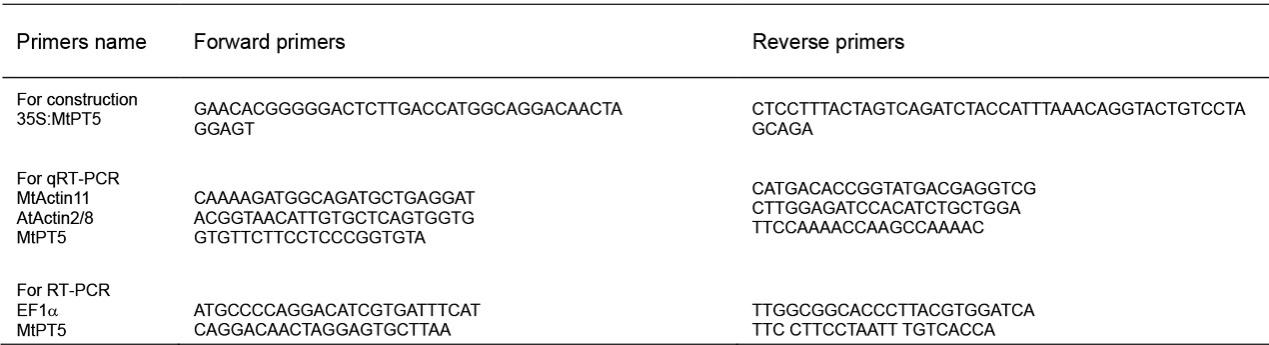

Supplement: Supplementary file 1 [file Data_Sheet_1.docx]
